# Nicotine protects astrocytes expressing alpha-synuclein against aminochrome cytotoxicity: Implications for Parkinson’s disease

**DOI:** 10.21203/rs.3.rs-9410157/v1

**Published:** 2026-05-09

**Authors:** Érica Novaes Soares, Cynthia Silva Bartolomeo, Tiago Nicoliche, Irlã Santos Lima, Lucas Matheus Gonçalves de Oliveira, Gabriel de Jesus Ferrolho, Rafaela Brito Oliveira, Roberta Sessa Stilhano, Silvia Lima Costa, Yousef Tizabi, Rodrigo Portes Ureshino, Victor Diogenes Amaral da Silva

**Affiliations:** Federal University of Bahia; Faculty of Medical Sciences of Santa Casa de São Paulo, , Brazil.; Faculty of Medical Sciences of Santa Casa de São Paulo, , Brazil.; Federal University of Bahia; Federal University of Bahia; Federal University of Bahia; Federal University of São Paulo; Faculty of Medical Sciences of Santa Casa de São Paulo, , Brazil.; Federal University of Bahia; Howard University College of Medicine; Federal University of São Paulo; Federal University of Bahia

**Keywords:** Parkinson’s disease, aminochrome, astrocytes, alpha-synuclein, nicotine, neuroprotection

## Abstract

Astrocytes containing alpha-synuclein (α-Syn) are a cytopathological finding in *post-mortem* samples of patients with Parkinson’s disease (PD). Aminochrome, a subproduct of dopamine oxidation, can induce formation of neurotoxic α-Syn oligomers, astrocyte reactivity, and astrocyte cell death. Nicotine, on the other hand, has been shown to have a protective effect against aminochrome cytotoxicity in substantia nigra dopaminergic cells. However, whether nicotine can also protect against aminochrome toxicity in α-Syn-expressing astrocytes is not known. To address this question, we used the human glioblastoma U251 cells stably overexpressing mutant A53T/nYFP α-Syn, and the U251 wild-type cells as a negative control. The results showed that treatments with 10 μM nicotine, for 24 or 48 h, protected U251 cells containing mutant α-Syn against aminochrome-induced cytotoxicity. Cell viability was assessed by MTT, and cleaved Caspase-3 by immunofluorescence. The protective effect of nicotine was also associated with an increase in acidic organelles in U251 cells containing mutant α-Syn. Overall, the results of this study reinforce the pharmacological potential of nicotine as a protective agent against brain cell degeneration especially relevant to PD.

## Introduction

Parkinson’s disease (PD) is a neurodegenerative disease marked by a degeneration of dopaminergic neurons in the midbrain. As the substantia nigra neurons are the main producers of dopamine in the brain (Cheng et al. 2010), the direct consequence of the neurodegeneration of this brain’s area results in the onset of the classic motor symptoms, such as dyskinesia, muscle rigidity, postural instability, and tremors at rest; as well as the non-motor symptoms, such as olfactory and mood disorders (Cheng et al. 2010; Hughes et al. 1992; Ross et al. 2004; Sveinbjornsdottir 2016).

Alpha-synuclein (α-Syn) is a protein present in the cell cytoplasm (Perfeito and Rego 2013; Mullin e Schapira 2013) and is crucial for the tyrosine hydroxylase phosphorylation, thus having a role in the neuronal synthesis of dopamine (Drolet et al. 2006). The discovery of α-Syn aggregates as the main component of inclusion bodies in neurons and glial cells was an important step in understanding the molecular mechanisms of PD (Mullin and Schapira 2013; Guan et al. 2025).

In addition to the formation of neurotoxic α-Syn aggregates, other molecular and cellular alterations such as mitochondrial dysfunction (Exner et al. 2012), dysfunction in the protein degradation by defective ubiquitin-proteasome system (Hauser and Hastings 2013), autophagy dysfunction (Kalia et al. 2013), increase in oxidative stress (Martinez-Vicente and Vila 2008), endoplasmic reticulum stress, and neuroinflammation may be involved in the loss of neurons in PD (Mullin and Schapira 2013). All of these mechanisms may contribute to the cytotoxic effects of aminochrome, an orthoquinone precursor of neuromelanin, capable of forming adducts with α-Syn, and consequently, stabilizing and generating neurotoxic protofibrils (Sulzer et al. 2000; Zecca et al. 2002; Zecca et al. 2008; Segura-Aguilar et al. 2014; Conway et al. 2001; Norris et al. 2005; Huenchuguala et al. 2014; Muñoz et al. 2012b; Meléndez et al. 2019; Briceño et al. 2016; Santos et al. 2017; De Araújo et al. 2018; Herrero et al. 2015). Aminochrome can also induce dysfunction in the macro autophagy/lysosomal system in astrocytes, which are essential for mitochondrial function and cell survival (Huenchuguala et al. 2014, 2016, 2017).

Given that excessive α-Syn is found in brains of PD patients (Mullin and Schapira, 2013; Wakabayashi et al. 2020; Ozoran et al. 2023), and the suggestion that nicotinic acetylcholine receptors in glial cells contribute to the protective effects of nicotine in PD (Soares et al. 2024), this study was carried out to determine whether nicotine may also protect against aminochrome-induced toxicity in an astrocytic cell line that overexpresses mutant α-Syn.

## Methodology

### U251 culture and transfection

U251 cell line (Sigma-Aldrich) was cultured in Dulbecco’s modified Eagle’s medium with nutrient mixture F12 (DMEM-F12), with glucose at 2.7 g/L and supplemented with 10% fetal bovine serum (FBS) and 1% penicillin/streptomycin (Gibco/ Invitrogen). Cells were kept in a 37°C and 5% CO_2_ incubator.

U251 cells overexpressing A53T mutant α-Syn (referred to as alpha-syn^+^ cells) were generated by transducing the construct containing the sequence of pBABE-α-Syn-nYFP (a kind gift from Huda Zoghbi, Addgene plasmid # 92203) by lentivirus transfection, in a BSL-2 laboratory at Santa Casa de Sao Paulo. For this purpose, HEK cells were used as packaging cells. After seeding the cells, the plasmids for the psPax2 for packaging, M5 for formation of the envelope, as well as the plasmid of interest, pBABE-α-Syn-nYFP, and calcium chloride, which contributes to precipitation, were also added. The lentivirus concentrate was used to transfect U251 cells. Cells transduced with the construct were selected with the antibiotic Geneticin 418 (G418), at a concentration of 200 μg/ml (Merck). After seeding the cells, plasmids consisting of psPax2 for packaging, M5 for formation of the envelope, and pBABE-α-Syn-nYFP, the plasmid of interest, as well as calcium chloride, which contributes to precipitation, were added. The virus concentrate was used to transfect U251 cells.

### Synthesis of aminochrome

Aminochrome was prepared by the oxidation of dopamine using a catalytic reaction with tyrosinase and purified according to De Araújo et al (2023). After being separated from the tyrosinase in column (12.7 × 0.30mm) with balanced CM-Sephadex C-25 resin containing MES (sodium 2-(N-Morpholino) ethanesulfonic acid), aminochrome was eluded by adding 7 mL 25 mM MES, pH 6.0. Product purity was assessed by measuring the absorbance at 460 nm.

### Cell treatment with nicotine

For treatments with nicotine, we used (−) Nicotine hydrogen tartrate salt (Sigma Aldrich, SML1236–50 mg) as previously assessed by Aryal et al (2021). For cell treatment, serial dilutions were performed over a concentration range of 0.1 μM to 100 μM. Treatments with nicotine occurred with or without aminochrome for a period of 24 h or 48 h.

### MTT viability assay

Cytotoxicity was determined using the 3-(4,5-dimethylthiazolyl-2)-2,5-diphenyltetrazolium bromide (MTT) assay. Cells were seeded in 96-well plates (0.5 × 10^4^ cells/cm^2^) and kept in an incubator with 5% CO_2_ at 37°C. After 24 hours, the cells were treated with nicotine at concentrations (0.0001, 0.001, 0.01, 0.1, 1, or 100 μg/ mL) for 24 or 48 hours. After the treatment period, the culture medium was replaced with MTT solution (5 mg/mL). The plate were then incubated for 3 h in the cell culture incubator. Subsequently, the culture medium containing MTT was removed, and 200 μL/well of DMSO was added for 15 min, followed by homogenization until the crystals were solubilized. Finally, the absorbance was measured at 590 nm in a spectrophotometer (Varioskan^™^ LUX multimode microplate reader).

### Trypan Blue assay test

For the Trypan blue assay test, U251 WT and transfected cells were seeded in an 8-well plate (Kasvi) at a density of 2.5 × 10^4^/cm^2^. After treatment with nicotine, the culture medium was removed from the cells, and the cells were washed 3x with PBS. Trypsin was added to release the cells from the plate. The cell suspension was centrifuged, and the supernatant was discarded. The cells were resuspended in a new medium. A volume of 45 μL was removed from the pool of cells and 15 μL of 0.4% trypan dye solution was added, followed by incubation for 10 min at 37°C. Afterwards, the cells were counted in a Neubauer chamber and classified as viable (unstained) or non-viable (stained).

### Immunofluorescence for Cleaved-caspase-3 and alpha-synuclein

Cleaved-caspase-3, a marker of apoptosis, and α-Syn are deeply linked in the pathogenesis of PD. Thus, abnormal accumulation and aggregation of α-Syn lead to the activation of caspase-3, which in turn causes neuronal death and further promotes α-Syn aggregation, creating a vicious cycle (Ma et al. 2018; Saramowicz et al. 2023).

U251 WT and transfected cells were seeded in chamber plates of 8 wells (Nunc Lab-Tek II Chamber Slide System, RS glass, Thermo Fisher Scientific) at a density of 1 × 10^4^/cm^2^. After treatments, cultures were washed three times with PBS at pH 7.4 and fixed with ice-cold methanol for 10 min. The boards were left to dry at room temperature. Cultures were rehydrated with PBS and permeabilized with PBS-T. Nonspecific binding of antibody was blocked by incubating the plates with 3% serum albumin (goat serum) in PBS. Cell cultures were then incubated with rabbit polyclonal antibody against cleaved Caspase-3 (1:10, Sigma-Aldrich, AB3623), an apoptosis marker. or with rabbit polyclonal antibody against alpha-synuclein (1:1,000, Invitrogen, Thermo Fisher, PA5–16738). Primary antibodies were diluted in PBS/BSA (1%). Cultures fixed and incubated with the solution were kept in a humid chamber at 4°C overnight. The next day, cells were washed 3 times with PBS and then incubated with a solution containing sheep anti-rabbit IgG secondary antibodies, fluorochrome-conjugated Alexa Fluor 594 (1:500, Life Technologies) diluted in PBS. Primary antibodies were diluted in PBS/BSA (1%). Fixed cultures incubated with the solution were kept in a humid chamber at 4°C overnight. The next day, cells were washed 3 times with PBS and then incubated with a solution containing sheep anti-rabbit IgG secondary antibodies, fluorochrome-conjugated Alexa Fluor 594 (1:500, Life Technologies), diluted in PBS. Cultures were washed 3 times with PBS and photographed immediately. To mount the slides and preserve fluorescence, a liquid mounting medium containing n-propyl gallate was used. The cultures were then photographed with a fluorescence microscope (Leica DMIL Led Microscope Fluo and Leica DFC7000 T Camera with Leica Apllication Suite software and module LAS Overlay for fluorescence, or on a confocal microscope (ZEISS LSM 880), courtesy of the Biology Institute of the Federal University of Bahia.

### Statistical analysis of data

For statistical analysis, one-way analysis of variance (ANOVA) followed by the Newman–Keuls post hoc test was used. Values of p ≤ 0.05 were considered statistically significant. All analyses were performed in at least three independent experiments.

## Results

### Effect of Nicotine on Wild-type U251

**Figure 1:**
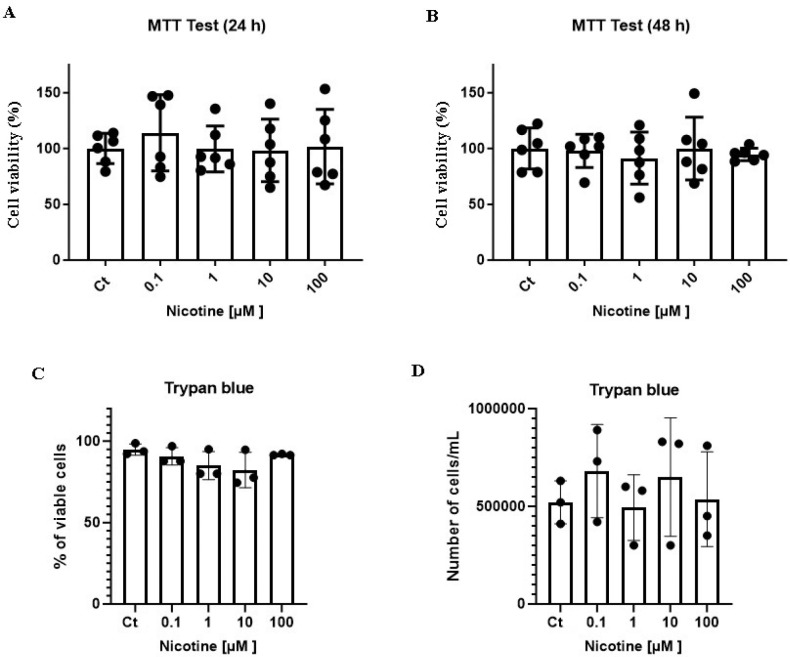
Treatment with 0.1 – 100 μM nicotine for 24 h or 48 h did not induce cytotoxicity in U251 wild-type cells. In A and B, U251 cells were treated with different concentrations of nicotine (ranging from 0.1 to 100 μM) or fresh medium in the control group, for 24 h (A) or 48 h (B). After treatment, the cell viability was evaluated using MTT test. In C and D, U251 cells were treated with different concentrations of nicotine (ranging from 0.1 to 100 μM) or fresh medium in the control group, for 48 h. After treatment, cell membrane integrity (C) and the total number of cells (viable and non-viable) (D) were analyzed using Trypan blue staining. Data were tested for significance using one-way analysis of variance (ANOVA) followed by the Newman–Keuls post hoc test (n = 3).

MTT test and Trypan blue staining revealed that treatment with nicotine 0.1–100 μM for 24 h or 48 h did not induce a change in the dehydrogenase activity (Fig. 1A and B), cell membrane integrity (Fig. 1C), or the number of cells, which estimates cell viability, when compared with the control group (Fig. 1C and D).

### Nicotine increases the dehydrogenase activity in U251 alpha-syn^+^ cells

**Figure 2:**
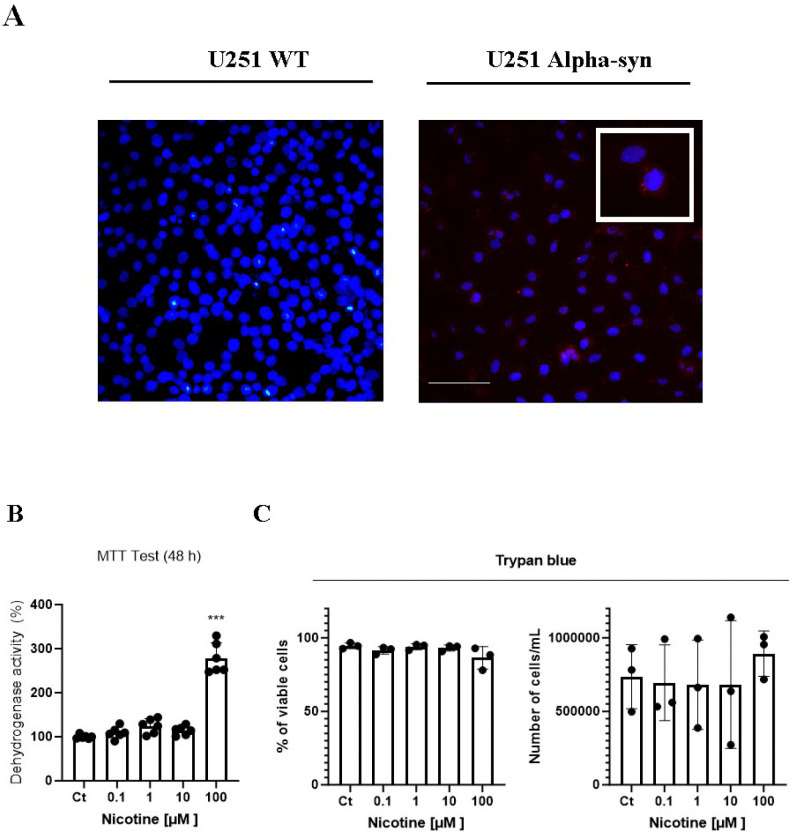
Treatment with 0.1 – 10 μM nicotine for 48 h did not induce cytotoxicity in U251 α-Syn+ cells. Treatment with 100 μM nicotine, however, increased the dehydrogenase activity in U251 α-Syn+ cells. In A, immunofluorescence images show U151-transfected cells expressing α-Syn in the cytoplasm, whereas wild-type cells did not express α-Syn. In B, U251 α-Syn^+^ cells were treated with different concentrations of nicotine (ranging from 0.1 to 100 μM) or fresh medium in the control group. Obj. 20X. Scale bar = 200 μm. After treatment, dehydrogenase activity was evaluated using the MTT test. Data were tested for significance using one-way analysis of variance (ANOVA) followed by the Newman–Keuls post hoc test (n = 6). The symbol *** represents p-value ≤ 0.001. In C, U251 α-Syn^+^ cells were treated with different concentrations of nicotine (ranging from 0.1 to 100 μM) or fresh medium in the control group, for 48 h. After treatment, the cell membrane integrity and the total number of cells (viable and non-viable) were analyzed using Trypan blue staining. Data were tested for significance using one-way analysis of variance (ANOVA) followed by the Newman–Keuls post hoc test (n = 3).

Immunofluorescence revealed that U251 α-Syn+ cells presented a diffuse cytoplasmic expression of α-Syn (Fig. 2A); whereas α-Syn expression was not detected in Wild-type cells. MTT test showed that treatment with 0.1–10 μM nicotine for 48 h did not change the dehydrogenase activity in U251 α-Syn+ cells (Fig. 2B). However, treatment with 100 μM nicotine for 48 h induced an increase in the dehydrogenase activity (278.4 ± 34.6, p ≤ 0.001), when compared with the control group (100 ± 4.5) (Fig. 2B).

Trypan blue staining revealed that treatment with nicotine at 0.1 – 100 μM for 48 h did not alter cell membrane integrity or viability compared with the control group (Fig. 2C).

### Nicotine protects U251 α-Syn ^+^ cells against aminochrome-induced damage

**Figure 3:**
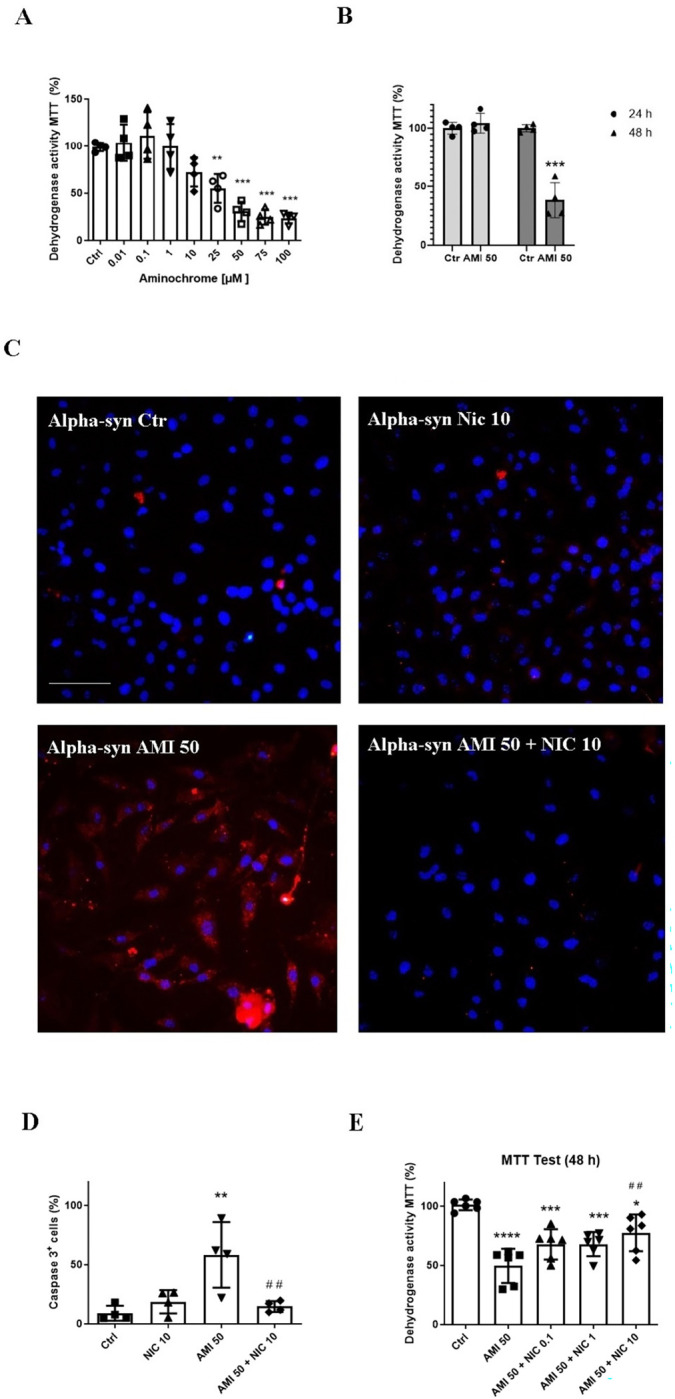
Treatment with 10 μM nicotine protects α-Syn^+^ U251 cells against aminochrome-induced cytotoxicity. In panel A, U251 α-Syn^+^ cells were treated with different concentrations of aminochrome (ranging from 0.01 to 100 μM) or with fresh medium in the control group for 48 h. In B, α-Syn^+^ U251 cells were treated with 50 μM aminochrome or with fresh medium (control) for 24 h or 48 h. In both panels A and B, the dehydrogenase activity was evaluated using the MTT assay. Significance effects were evaluated using one-way analysis of variance (ANOVA) followed by the Newman–Keuls post hoc test (n = 4). In panel C, images of α-Syn^+^ U251 cells processed by immunofluorescence for cleaved caspase 3 are shown in red and for DAPI-stained nucleus are shown in blue. Obj 20x scale = 200μm. In panel D, the percentage of caspase-3-positive cells/Dapi-stained nuclei was counted using the ImageJ program. Data represent the mean ± SD of caspase 3 positive cells. The significance was analysed using ANOVA followed by the Newman–Keuls post hoc test (n = 3); *p ≤ 0.05. In panel E, α-Syn^+^ U251 cells were treated with 50 μM aminochrome and/or 10 μM nicotine or with fresh medium (control) for 48 h. Dehydrogenase activity was evaluated using the MTT assay. One-way analysis of variance (ANOVA) followed by the Newman–Keuls post hoc test was applied (n = 4). Symbols represent **** p≤ 0.0001; *** p≤ 0.0005; *p≤ 0.05, compared with the control group; and ## *** p≤ 0.001, compared with the aminochrome group.

Before assessing the protective effect of nicotine against aminochrome-induced damage in U251 α-Syn^+^ cells, time and concentration curves of cytotoxic effect of aminochrome were evaluated using the MTT assay. It was observed that cultures treated with aminochrome for 48 h presented a reduction in cell viability when exposed to concentrations of 25 μM (55 ± 15%), 50 μM (30 ± 10%), 75 μM (25 ± 8%), and 100 μM (24 ± 6%), when compared with the control group (99 ± 4%) (Fig. 3A). Since treatment with the 50 μM aminochrome for 48 h resulted in significant toxicity, to the cells, we chose this concentration and time point in all subsequent studies (Fig. 3B).

Whereas the MTT assay did not show a decrease in the dehydrogenase activity in α-Syn^+^ U251cells treated with 50 μM aminochrome for 24 h (Fig. 3B), the immunofluorescence showed a significant increase in the percentage of cleaved-caspase 3-positive cells induced by these treatment conditions (58.3 ± 28%) compared with the control group (9.0 ± 6.3%; p = 0.0025) (Fig. 3C and D). On the other hand, treatment with 10 μM nicotine alone or in combination with aminochrome for 24 h did not change the percentage of cleaved-caspase 3-positive cells, when compared with the control group. The percentage of cleaved-caspase 3-positive cells in the group treated with the combination of aminochrome and nicotine (14.7 ± 4.5%) was lower than that detected in the group treated with aminochrome alone (58.3 ± 28%; p = 0.0063), showing a protective effect of nicotine against aminochrome toxicity (Figs. 3C and D).

The protective effect of 10 μM nicotine in α-Syn^+^ U251 cells against aminochrome toxicity was also evident in the MTT assay (Fig. 3E).

## Discussion

Although the etiology of PD remains an enigma, cumulative evidence suggests that accumulation of α-Syn plays a major role as degeneration of dopaminergic neurons in the substantia nigra is underscored by the presence of Lewy bodies, which are primarily composed α-Syn aggregates (Guan et al. 2025). α-Syn is not only expressed in the neurons but also in the astrocytes, which normally play a neuroprotective role but can shift to a toxic role, whereby α-Syn accumulation and neuroinflammation are manifested (Hindeya Gebreyesus and Gebrehiwot Gebremichael 2020; Nordengen and Morland 2024). Thus, considerable effort is devoted to exploring potential targeting of astrocytes in PD.

Aminochrome-induced toxicity in cellular models has been used to identify novel neuroprotectants. Curiously, in this model as well as several other in-vitro and in-vivo models of PD, protective effects of nicotine have been verified, suggesting its potential clinical application (Muñoz et al. 2012; Soares et al. 2024). The results of our study support this notion as we show that nicotine can also protect against aminochrome-induced toxicity in astrocytes expressing α-Syn.

That nicotine may have beneficial effects in PD was highlighted by several epidemiological studies showing that the incidence of PD is lower in smokers, although smoking has been strongly linked to premature death (Kumari et al. 2025; Lin et al. 2025). Nicotine’s mechanism of action as well as its therapeutic potentials not only in PD but other neurodegenerative/neuropsychiatric disorders has been amply reviewed (Quik et al. 2019; Tizabi et al. 2021; Soares et al. 2024; Delgado et al. 2025; ElNebrisi et al. 2025; Lin et al. 2025). It is now believed that nicotine, via activation of several nicotinic receptor (nAChR) subtypes, including alpha4-beta2 and alpha7 imparts substantial protective effects including apoptosis inhibition, reduction in metal ion (e.g., iron, copper, or zinc) overload, maintenance of calcium homeostasis, mitochondrial respiratory chain function as well as antioxidant and antiinflammatory effects (Delgado et al. 2025).

We used both MTT assay, which measures cell metabolism by assessing dehydrogenase activity (Slater et al. 1963), and the Trypan Blue test, which assesses the cell membrane’s selective permeability (Strober 2015). Both are considered classical cytotoxicity assays but can also indirectly indicate cell proliferation in culture. However, since nicotine did not affect Trypan Blue test, cell proliferation effect cannot be claimed. Nonetheless, our finding of protective effects of nicotine against aminochrome toxicity in astrocytes is consistent with reports of increased astrocyte metabolism and reactivity by nicotine (Sewell and Cray, 2025). Interestingly, these effects of nicotine were shown to be due to α7 and α4β2 nAChR activation (Aryal et al. 2021; Sewell and Cray, 2025).

Caspase-3 is a cysteine-aspartic acid protease, which, after being cleaved by initiator caspases during apoptotic flow, becomes key as an effector in cell apoptosis (Asadi et al. 2022). Previous studies demonstrate that aminochrome-induced mitochondrial damage triggers apoptosis in substantia nigra cells (Paris et al. 2011). Here, we observed that cell death induced by aminochrome exposure is preceded by an increase in caspase 3, which was inhibited by nicotine treatment, further supporting the antiapoptotic activity of nicotine. Antiapoptotic activity of nicotine have also been observed against chemotherapeutic drugs (Dasgupta et al. 2006).

## Conclusion

This study shows that nicotine may protect against aminochrome-induced toxicity in α-Syn expressing astrocytes. The results also suggest an anti-apoptotic effect of nicotine. Altogether, the findings support the potential utility of nicotine in PD.

## Data Availability

Mean and SD data are contained within the article
